# Proteasome inhibition protects blood–brain barrier P-glycoprotein and lowers Aβ brain levels in an Alzheimer’s disease model

**DOI:** 10.1186/s12987-023-00470-z

**Published:** 2023-10-06

**Authors:** Milica Vulin, Yu Zhong, Bryan J. Maloney, Björn Bauer, Anika M. S. Hartz

**Affiliations:** 1 Sanders-Brown Center On Aging, University of Kentucky, Lexington, KY USA; 2 Department of Pharmaceutical Sciences, College of Pharmacy, University of Kentucky, Lexington, KY USA; 3 Department of Pharmacology and Nutritional Sciences, University of Kentucky, 124 Healthy Kentucky Research Building 760 Press Avenue, Lexington, KY 40508 USA

**Keywords:** Blood–Brain Barrier, P-glycoprotein, Alzheimer’s disease, Ubiquitin–proteasome system, Amyloid beta, Clearance

## Abstract

**Background:**

Loss of P-glycoprotein (P-gp) at the blood–brain barrier contributes to amyloid-β (Aβ) brain accumulation in Alzheimer’s disease (AD). Using transgenic human amyloid precursor protein (hAPP)-overexpressing mice (Tg2576), we previously showed that Aβ triggers P-gp loss by activating the ubiquitin–proteasome pathway, which leads to P-gp degradation. Furthermore, we showed that inhibiting the ubiquitin-activating enzyme (E1) prevents P-gp loss and lowers Aβ accumulation in the brain of hAPP mice. Based on these data, we hypothesized that repurposing the FDA-approved proteasome inhibitor, bortezomib (Velcade^®^; BTZ), protects blood–brain barrier P-gp from degradation in hAPP mice in vivo.

**Methods:**

We treated hAPP mice with the proteasome inhibitor BTZ or a combination of BTZ with the P-gp inhibitor cyclosporin A (CSA) for 2 weeks. Vehicle-treated wild-type (WT) mice were used as a reference for normal P-gp protein expression and transport activity. In addition, we used the opioid receptor agonist loperamide as a P-gp substrate in tail flick assays to indirectly assess P-gp transport activity at the blood–brain barrier in vivo. We also determined P-gp protein expression by Western blotting, measured P-gp transport activity levels in isolated brain capillaries with live cell confocal imaging and assessed Aβ plasma and brain levels with ELISA.

**Results:**

We found that 2-week BTZ treatment of hAPP mice restored P-gp protein expression and transport activity in brain capillaries to levels found in WT mice. We also observed that hAPP mice displayed significant loperamide-induced central antinociception compared to WT mice indicating impaired P-gp transport activity at the blood–brain barrier of hAPP mice in vivo. Furthermore, BTZ treatment prevented loperamide-induced antinociception suggesting BTZ protected P-gp loss in hAPP mice. Further, BTZ-treated hAPP mice had lower Aβ40 and Aβ42 brain levels compared to vehicle-treated hAPP mice.

**Conclusions:**

Our data indicate that BTZ protects P-gp from proteasomal degradation in hAPP mice, which helps to reduce Aβ brain levels. Our data suggest that the proteasome system could be exploited for a novel therapeutic strategy in AD, particularly since increasing Aβ transport across the blood–brain barrier may prove an effective treatment for patients.

## Introduction

Impaired amyloid-β (Aβ) brain clearance across the blood–brain barrier is recognized as one key factor that contributes to Aβ brain accumulation in Alzheimer's disease (AD) [1–5]. Furthermore, data from several studies show that the ATP-binding cassette transporter P-glycoprotein (P-gp) contributes to Aβ transport from the brain across the brain capillary endothelium into the blood, suggesting P-gp is critical for Aβ clearance [6–13]. In AD, however, loss of P-gp at the blood–brain barrier has been documented in different mouse AD models and, more importantly, is also well-documented in brain capillaries isolated from samples of AD patients compared to samples from age-matched cognitively healthy individuals [14–23]. However, the mechanism underlying P-gp loss at the blood–brain barrier of AD patients remains poorly understood, and a therapeutic strategy to rescue and/or protect P-gp is not available.

In this regard, we previously showed in transgenic human amyloid precursor protein (hAPP)-overexpressing mice that activating the pregnane X receptor with pregnenolone-16-carbonitrile induces P-gp protein expression and transport activity levels, which restores Aβ clearance and reduces brain Aβ levels [9]. We also demonstrated that Aβ40 triggers P-gp ubiquitination, which was followed by transporter internalization and degradation via the ubiquitin–proteasome system [24–26]. Further, exposing freshly isolated rat brain capillaries to Aβ40 decreased P-gp activity levels and increased ubiquitinated P-gp levels; these effects were reversed when the proteasome was blocked [25]. Consistent with these findings, we also showed reduced P-gp protein levels and increased P-gp ubiquitination levels in isolated brain capillaries from AD patients compared to capillaries from cognitively normal individuals [26]. Moreover, inhibiting the ubiquitin-activating enzyme (E1) prevented P-gp degradation and subsequent Aβ brain accumulation in hAPP mice [26]. Based on these results, we hypothesized that the ubiquitin–proteasome system degrades blood–brain barrier P-gp in AD, thus protecting P-gp from degradation improves Aβ clearance and lowers Aβ brain levels. Therefore, in the present study, we explored targeting the proteasome with the FDA-approved proteasome inhibitor bortezomib (Velcade^®^, BTZ) to block P-gp proteasomal degradation in an AD mouse model in vivo.

## Methods

### Experimental design and statistical analysis

Sample and group sizes (animal numbers, brain capillary numbers, tissue sample numbers) are based on power analyses of preliminary data and previously published work [9, 24–27]. Sample size and repetition number (n) are provided under the Results and the respective figure legends.

Results are presented as mean ± SEM. Several animals, particularly of the P-gp KO or hAPP + BTZ + CSA groups, did not have a tail flick by the ten-minute stop point. Analysis, therefore, required a censored regression model [28, 29]. Latency in minutes of tail flick response was analyzed by censored regression with animal as clustering factor to account for repeat measurements of the same animal [30]. Non-linear time effect was modeled by natural splines, testing up to degrees of freedom in the function. In all cases, the model Latency ~ Time + Treatment + Time × Treatment was used, with second-order Akaike Information Criterion (AICc) to compare different spline degrees of freedom. Other responses (such as AUC) were modeled with generalized linear models of outcome measures vs. treatment, followed by analysis of deviance (ANOVA). ANOVA was followed by estimating marginal means [31] for pairwise comparisons within the models, adjusted by false discovery rate for multiple comparisons. All estimates were done with the R statistical environment. Differences were statistically significant at *p* ≤ 0.05. Pairwise differences, when explicitly stated, were expressed as percent change and Hedges’ *g* standardized effect size. When data included censored values, “mean” values were, perforce, estimated from models. Time-based modeling (tail-flick assay) was performed using B-splines for time to account for non-linear responses.

### Chemicals

Antibodies to human Aβ40 (ab12265; RRID:AB_298985), human Aβ42 (ab12267; RRID:AB_298987), and β-actin (ab8226; RRID: AB_306371) were purchased from Abcam (Cambridge, MA, USA). C219 antibody against P-gp was from ThermoFisher (MA126528; RRID:AB_795165; Waltham, MA, USA). Complete^™^ protease inhibitor was obtained from Roche (Mannheim, Germany). Fluorescein-β-amyloid1-42 (FL-hAβ42) was purchased from rPeptide (Bogart, GA, USA). [N-ε (4-nitrobenzofurazan-7-yl)-D-Lys8]-cyclosporine A (NBD-CSA) was custom-synthesized by R. Wenger (Basel, Switzerland) [32]. PSC833 was a gift from Novartis (Basel, Switzerland). Bortezomib was obtained from Selleckchem (Houston, TX, USA). Cyclosporin A was purchased from Tocris Bioscience (Bristol, United Kingdom). Dulbecco’s phosphate-buffered saline (DPBS) with 0.9 mM Ca^2+^ and 0.5 mM Mg^2+^ was from HyClone (Logan, UT, USA). CelLytic™ M, Ficoll® PM 400, bovine serum albumin, and all other chemicals were from Sigma (St. Louis, MO, USA).

### Animals

All animal experiments were approved by the Institutional Animal Care and Use Committee of the University of Kentucky (protocol#: 2014–1233, PI: Hartz, AMS) and carried out in accordance with AAALAC regulations, the US Department of Agriculture Animal Welfare Act, and the Guide for the Care and Use of Laboratory Animals of the NIH.

Male P-gp knockout (KO) mice (CF-1 strain; CF1-Abcb1amds – PGP) and corresponding male CF-1 wild-type (WT) mice were obtained from Charles River Laboratories (Wilmington, MA, USA). Mice were 9 weeks old with an average body weight of 36.3 ± 2.8 g for P-gp KO and 33.4 ± 1.4 g for WT mice. Male transgenic human amyloid precursor protein (hAPP)-overexpressing mice (Tg2576 model; 129S6.Cg-Tg(APP_SWE_)2576Kha) and corresponding male WT 129S6 mice were purchased from Taconic Farms (Germantown, NY, USA). Mice were 10–11 weeks old with an average body weight of 27.5 ± 1.8 g for WT mice and 27.8 ± 2.2 g for transgenic hAPP mice.

All animals were single-housed and kept under controlled environmental conditions (21 °C; 51–62% relative humidity; 12-h light/dark cycle) using an EcoFlo Allentown ventilation system (Allentown Inc., Allentown, NJ, USA). Animals were monitored daily and had free access to tap water and Harlan Teklad Chow 2918 rodent feed (Harlan Laboratories Inc., Indianapolis, IN, USA). Upon arrival in the animal facility, animals were allowed to acclimate for at least 7 days before experiments.

### Bortezomib dosing

**Fig. 1:**
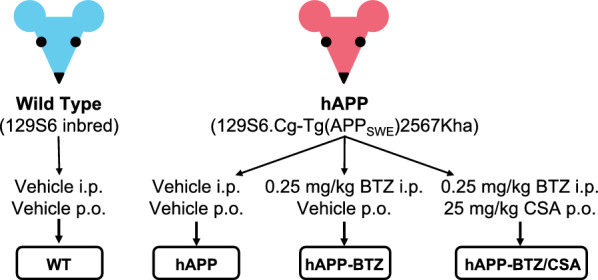
Experimental Design: Mouse Models and BTZ Treatment. Schematic diagram of mouse genotypes and BTZ treatment: Wild type (WT) mice treated with vehicle, administered both i.p. and p.o. and hAPP mice treated according to one of three regimens: vehicle (i.p. and p.o.); BTZ (i.p.) and vehicle (p.o.); or BTZ (i.p.) and CSA (p.o.). After the treatment period, animals were euthanized, and tissue samples were collected

Animals were randomly divided into four groups with n = 25 mice per group (Fig. 1). Group 1: WT mice; group 2: hAPP mice; group 3: hAPP mice dosed with bortezomib (BTZ); group 4: hAPP mice dosed with BTZ and the P-gp inhibitor cyclosporine A (CSA; FDA-approved). Mice were dosed for 14 days; mice in groups 1 and 2 received vehicle (5% PEG200; 0.5% Tween80; 94.5% saline) i.p. injections on days 1, 4, 7, 10, and 13 (every third day) and were given vehicle (10% PEG200; 20% Cremophor EL; 70% saline) via oral gavage (p.o.) on the two days in between i.p. injections. Mice in group 3 received 0.25 mg/kg BTZ (i.p.) on days 1, 4, 7, 10, and 13 and vehicle (p.o.) on the days between the BTZ doses. Mice in group 4 received 0.25 mg/kg BTZ on days 1, 4, 7, 10, and 13 and were given 25 mg/kg CSA by oral gavage on the two days in between BTZ dosing (Table 1). In addition, animal weight was monitored every three days. The dosing regimen was based on our previously published studies and preliminary data using BTZ [24–26].

**Table 1:**
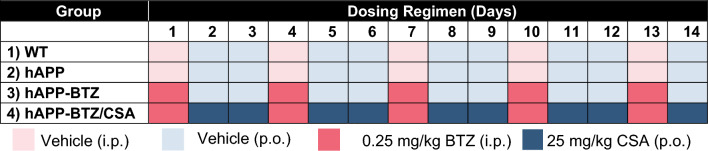
Treatment schedule

### Tail flick assay

Tail flick assays were conducted with n = 10 mice per group. Starting 7 days before tail flick measurements, mice were handled and restrained daily for animals to adapt to the procedure. After treating mice for two weeks with BTZ, BTZ-CSA, and/or vehicle, tail flick assays were conducted using a LE 7106 Light Beam analgesia meter (Harvard Apparatus, Holliston, MA, USA). Two hours before loperamide injections, mice in group 4 received 50 mg/kg CSA (i.p.); mice in the other groups received vehicle (i.p.). Next, mice were dosed with 5 mg/kg loperamide (s.c.), which was immediately followed by tail flick tests. Radiant heat was applied to a mouse’s tail, and the time from the onset of heat exposure until the mouse flicked its tail due to thermal nociception was defined as tail flick latency time. Tail flick latency time was measured automatically, and the intensity of the thermal stimulus was adjusted to produce baseline latencies of 2–4 s. Cutoff time was set to 10 s to avoid tissue damage to the animal’s tail, at which point latency was recorded as “10 censored” and analyzed accordingly. Tail flick latency times were measured at 0, 30, 60, 120, 180, and 360 min after loperamide dosing and determined once per animal per time point. To determine the overall latency, the area under the curve (AUC) was calculated with the trapezoidal method using the following equation:

$$AUC = \,\sum\nolimits_{i = 0}^{n} {\frac{{\left( {Lat_{n} + Lat} \right)}}{2}} \times \,(t_{n} - t_{n - 1} ),$$

where *Lat (Latency)* was normalized by subtracting minimum latency for that animal and *t* (time in [s]) was normalized by dividing by 40, to produce a maximum time of 9, similar to the maximum *Lat* value of 8.8, to prevent domination of AUC by “minutes” over “latency” due to highly disparate ranges. Calculating derived outcomes can be complex. For our purposes, we adopted a rule of thumb that if over 50% of an animal's latencies were censored (10 min), then the AUC for that animal was also censored.

### Blood and tissue collection

After the 14-day treatment period and the tail flick assays, mice were euthanized by CO_2_ asphyxiation and decapitated; brain tissue and trunk blood were collected from each animal. Blood was stored in heparinized tubes (BD Microtainer^®^ PSI Tubes with Lithium Heparin (#365985; BD Biosciences, Franklin Lakes, NJ, USA), and plasma was extracted from blood by centrifugation at 5000 *g* for 5 min at 4 °C. Plasma and brain tissue samples were snap-frozen in liquid nitrogen at collection and stored at − 80 °C for later analysis.

### Brain capillary isolation

Brain capillaries were isolated as previously described [33–35]. In brief, whole brains were dissected, meninges, white matter and choroid plexus were removed. The remaining cortex tissue was homogenized in 4 °C cold DPBS buffer (2.7 mM KCl, 1.46 mM KH_2_PO_4_, 136.9 mM NaCl, 8.1 mM Na_2_HPO_4_, 5 mM D-glucose, 1 mM sodium pyruvate, pH 7.4). Ficoll® was added to the brain homogenate to a final concentration of 15%, and the Ficoll^®^/brain mixture was centrifuged at 5800 *g* for 20 min at 4 °C. The pellet was resuspended in 1% BSA/DPBS, and the capillary suspension was passed over a glass bead column to purify capillaries from nonspecific debris and red blood cells. Capillaries adhering to the glass beads were collected by gentle agitation in 1% BSA/DPBS, washed with BSA-free DPBS, and used for experiments.

### Capillary Crude Membrane Isolation

Capillary crude membranes were isolated as previously published [9, 25, 35]. First, isolated brain capillaries were lysed in CelLytic^™^ M lysis buffer containing Complete^™^ protease inhibitor and centrifuged at 10,000 g for 15 min at 4 °C. Next, the supernatant was centrifuged at 100,000 g for 90 min at 4 °C. Finally, the resulting capillary crude membrane pellet was resuspended and stored at − 80 °C.

### P-glycoprotein Transport Assay

P-gp transport activity was determined by incubating freshly isolated brain capillaries with the P-gp-specific fluorescent substrate NBD-CSA (2 μM) for 1 h at room temperature [9, 36–38]. For Aβ transport studies, brain capillaries were incubated for 1 h at room temperature with 5 μM FL-hAβ42 in DPBS containing 5 mM D-glucose and 1 mM sodium pyruvate (pH 7.4) [7, 9, 39]. Per treatment group, images of 7–15 capillaries were acquired using a Zeiss LSM 710 inverted confocal microscope (40 × 1.2 NA water immersion objective, 488 nm line of argon laser, Carl Zeiss Inc., Thornwood, NY, USA). Isolated capillaries adhering to the glass cover slip were randomly selected. To ensure consistency of image collection all imaging parameters (i.e., laser power, gain, pinhole, zoom, image format, imaging speed as well as line and frame average) across groups were kept the same across experiments. Images were analyzed by quantitating luminal NBD-CSA and FL-hAβ42 fluorescence using ImageJ software v1.48. The fluorescent images of each capillary were digitally magnified, and three adjacent luminal areas were selected. The areas were chosen to encompass the entire length of the capillary that was in focus. Average pixel intensity for each capillary was calculated. The value used for that capillary was the means of all selected areas. Specific luminal NBD-CSA and FL-hAβ42 fluorescence was calculated as the difference between total luminal fluorescence and fluorescence in the presence of the P-gp-specific inhibitor PSC833 (5 μM) [9, 36–38].

### Aβ immunostaining in brain capillaries

Isolated brain capillaries were fixed with 3% paraformaldehyde/0.25% glutaraldehyde for 30 min at room temperature. After washing with PBS, capillaries were permeabilized with 0.5% Triton X-100 for 30 min and washed with PBS. Next, capillaries were blocked with 1% BSA/PBS for 60 min and incubated overnight at 4 °C with a 1:500 dilution of polyclonal antibody against hAβ40 (0.5 µg/ml; ab12265; RRID: AB_298985) or hAβ42 (0.5 µg/ml ab12267; RRID:AB_298987). After primary antibody incubation, capillaries were washed and incubated with Alexa-Fluor 488-conjugated secondary IgG (0.75 µg/ml; Invitrogen, Carlsbad, CA, USA) for 1 h at 37 °C. Nuclei were counterstained with 2 µg/ml DAPI. Negative controls for each treatment were processed without primary antibody and showed negligible background fluorescence (data not shown). Immunofluorescence was visualized using a Zeiss LSM 710 inverted confocal microscope (40 × 1.2 NA water immersion objective, 488 nm line of argon laser, Carl Zeiss Inc., Thornwood, NY, USA). Per treatment group, confocal images of 10 capillaries were acquired. Aβ membrane immunofluorescence for each capillary was quantitated using ImageJ software v1.48. As previously described [40], a 10 × 10 grid was superimposed on each image, and fluorescence measurements of capillary membranes were taken between intersecting grid lines. Fluorescence intensity for each capillary was the mean of 6 measurements from each capillary.

### Western blotting

Protein expression levels in brain capillary crude membranes were analyzed by Western blotting as previously described [9, 25, 36]). Western blotting was performed using the Invitrogen NuPage™ Bis–Tris electrophoresis and blotting system (Invitrogen, Carlsbad, CA, USA). After electrophoresis and protein transfer, membranes were blocked and incubated overnight with the primary antibody as indicated (P-gp: 1:100 (1 µg/ml; MA126528; RRID:AB_795165; ThermoFisher, Waltham, MA, USA); β-actin: 1:1000 (1 µg/ml; ab8226; RRID: AB_306371, Abcam, Cambridge, MA, USA); hAβ40: 1:500 (1 µg/ml; ab12265; RRID:AB_298985, Abcam, Cambridge, MA, USA); hAβ42: 1:500 (1 µg/ml; ab12267; RRID:AB_298987, Abcam, Cambridge, MA, USA). Next, membranes were washed and incubated for 1 h with horseradish peroxidase-conjugated ImmunoPure^®^ secondary IgG (1:10,000; 0.25 µg/ml; Pierce, Rockford, IL, USA). Proteins were detected with SuperSignal^®^ West Pico Chemiluminescent Substrate (Pierce, Rockford, IL, USA) and visualized with a BioRad Gel Doc 2000™ gel documentation system (BioRad, Hercules, CA, USA).

### Aβ40 and Aβ42 ELISA

Aβ40 and Aβ42 levels in plasma and brain samples were determined by ELISA (KHB3482; KHB3442) according to the manufacturer’s protocol (Invitrogen, Carlsbad, CA, USA). Aβ40 and Aβ42 were extracted from brain tissue by homogenizing the samples with Tris–HCl buffer containing 5 M guanidine-HCl. Samples were diluted 1:20 in DPBS buffer containing 5% BSA and 0.03% Tween-20 and centrifuged at 16,000 *g* for 20 min at 4 °C. The supernatant was collected and diluted 1:1 before adding it to ELISA plates.

### Proteasome activity assay

Following the manufacturer's protocol, protein activity in brain tissue lysate was determined using a proteasome activity assay kit (ab107921, Abcam, Waltham, MA, USA). First, 20 mg of brain tissue or isolated brain capillaries were homogenized in 200 µl of 0.5% NP-40 lysis buffer using a hand-held motorized homogenizer (Kimble® Pellet Pestle® cordless motor homogenizer, 749450-0000, DWK Life Sciences, Millville, NJ, USA). Next, homogenized brain samples were centrifuged at 15,000 *g* for 15 min at 4 °C. Next, 30 µl of the supernatant was pipetted in duplicate onto a white 96-well microplate with clear bottom wells along with standards and a positive control. Proteasome inhibitor was added to one set of wells, assay buffer was added to the other set, and proteasome substrate was added to all wells.

The plate was incubated at 37 °C for 20 min while protected from light. Fluorescence was measured at Ex/Em = 350/440 nm using a Synergy^™^ H1 Hybrid Multi-Mode plate reader (BioTek, Winooski, VT, USA). The plate was again incubated at 37 °C for 30 min and protected from light, and fluorescence was remeasured using the same parameters. The standard curve was generated using different concentrations of a 7-amino-4-methylcoumarin-(AMC)-tagged peptide that releases highly fluorescent AMC when exposed to proteolytic activity. To determine AMC levels in the sample wells specifically generated by proteolytic activity, the change in relative fluorescence units (RFUs) between the two time points was plotted on the standard curve.

$$\Delta RFU \, = \, \left( {RFU2 \, - \, iRFU2} \right) \, - \, \left( {RFU1 \, - \, iRFU1} \right)$$

Proteasome activity is reported as pmol AMC/min × µl. Activity of proteasome in the samples were then calculated according to the following equation:

Proteasome activity = $$\left(\frac{B}{\left({T}_{2}- {T}_{1}\right)*V}\right)\times D$$

## Results

### Bortezomib effect on P-gp protein expression and transport activity in hAPP mice

We hypothesized that proteasome inhibition protects blood–brain barrier P-gp from proteasomal degradation in hAPP mice in vivo. We used the FDA-approved proteasome inhibitor bortezomib (BTZ, Velcade^®^) and dosed 10–11-week-old hAPP mice with 0.25 mg/kg BTZ (i.p.) every three days for a total of 14 days (Table 1**; **Fig. 1). A second group of mice received BTZ every three days, and on the two days between BTZ injections, these mice also received the P-gp inhibitor cyclosporin A (CSA; 25 mg/kg; p.o.). Mice receiving the BTZ/CSA combination were controls for P-gp transport activity. Wild Type (WT) and hAPP control mice received vehicle treatments (i.p.; p.o.) only.

**Fig. 2:**
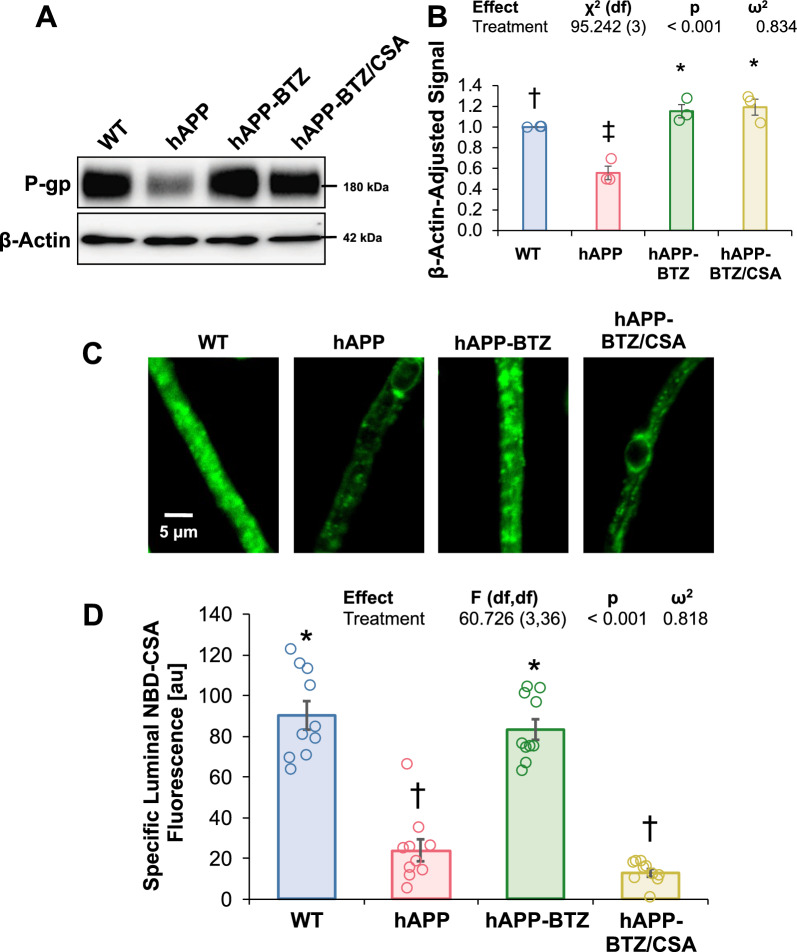
BTZ effect on P-gp protein expression and transport activity in brain capillaries isolated from hAPP mice. **A** Representative Western blot for P-gp showing bands for brain capillary membranes isolated from wild-type (WT), hAPP, hAPP treated with 0.25 mg/kg BTZ, and hAPP treated with a combination of BTZ/CSA (0.25/25 mg/kg). β-Actin was used as protein loading control. Tissue was pooled from all mice per treatment group (WT: n = 20 mice; hAPP: n = 18 mice; hAPP-BTZ: n = 20 mice, hAPP-BTZ/CSA: n = 19 mice). **B** Densitometric analysis of three western blots for P-gp levels. Blotting and probing were performed on three separate gels, as described in the main text. Data were analyzed by mixed-level generalized linear models to account for individual blot effects. Columns represent model-estimated marginal means (WT = 1), excluding random effects ± SEM for 3 blots. **C** Representative confocal images of isolated brain capillaries from WT, hAPP, hAPP-BTZ, and hAPP-BTZ/CSA mice showing luminal accumulation of the P-gp-specific substrate [N-ε (4-nitrobenzofurazan-7-yl)-D-Lys8]-cyclosporine A (NBD-CSA) after a 1-h incubation (steady state; 2 μM NBD-CSA). **D** Data obtained by image analysis using ImageJ. Specific NBD-CSA fluorescence is the difference between total luminal NBD-CSA fluorescence and NBD-CSA fluorescence in the presence of the P-gp-specific inhibitor PSC833 (5 μM). Columns represent mean ± SEM for 10 capillaries from one preparation (pooled tissue: WT: n = 20 mice; hAPP: n = 18 mice; hAPP-BTZ: n = 20 mice, hAPP-BTZ/CSA: n = 19 mice), as arbitrary fluorescence units (scale 0–255). The ω^2^ is an unbiased estimator of the coefficient of determination (*R*^2^) for an effect. Symbols indicate statistical categories at *p* ≤ 0.05 derived from pairwise comparisons adjusted for false discovery rate. Treatments sharing a symbol did not differ at *p* ≤ 0.05

After dosing mice for two weeks, we assessed P-gp protein and transport activity levels in isolated brain capillaries from each group. P-gp protein levels in brain capillaries isolated from BTZ-treated hAPP were comparable to those isolated from vehicle-treated WT mice (Fig. 2A). We made a similar observation in isolated capillaries from hAPP mice treated with the BTZ/CSA combination. To quantitate P-gp protein levels, we determined the optical density of WBs and normalized the data to β-actin (Fig. 2B). hAPP mice had significantly lower P-gp levels compared to WT mice (44% signal reduction, Hedges’ *g* = − 6.409). However, BTZ treatment protected P-gp in hAPP mice, resulting in P-gp levels that were even 15% higher than WT levels (Hedges’ *g* = 1.555). There was no significant difference in P-gp protein expression levels in capillaries from BTZ/CSA-treated hAPP mice compared to BTZ-treated hAPP mice (3% difference between BTZ/CSA and BTZ, Hedges’ *g* = 0.364).

In addition to P-gp protein levels, we also determined P-gp transport activity levels using an assay we previously established [9, 25, 26, 37]. In this assay, freshly isolated brain capillaries are incubated with 2 μM of the fluorescent P-gp-specific substrate NBD-cyclosporin A (NBD-CSA). After a 1-h incubation, brain capillaries undergo live-cell imaging using a confocal microscope (Fig. 2C**).** Luminal NBD-CSA accumulation is an indirect measure of P-gp transport activity and is assessed with quantitative image analysis. [9, 25, 26, 37]. Image analysis revealed that hAPP mice had significantly lower luminal NBD-CSA fluorescence compared to WT mice (Fig. 2D; 74% signal reduction; Hedges’ *g* = − 3.253). In contrast, treating hAPP mice with BTZ restored NBD-CSA fluorescence to nearly WT levels (8% signal reduction vs. WT; Hedges’ *g* = − 0.348). Compared to WT mice, luminal NBD-CSA fluorescence in brain capillaries from BTZ/CSA-treated hAPP mice was reduced by 86% and was indistinguishable from levels detected for vehicle-treated hAPP mice.

In summary, we found that treating hAPP mice with the proteasome inhibitor BTZ for 2 weeks protected P-gp resulting in P-gp protein expression and transport activity levels in brain capillaries comparable to those found in capillaries from WT mice.

### Bortezomib effect on P-gp-mediated hAβ transport

**Fig. 3:**
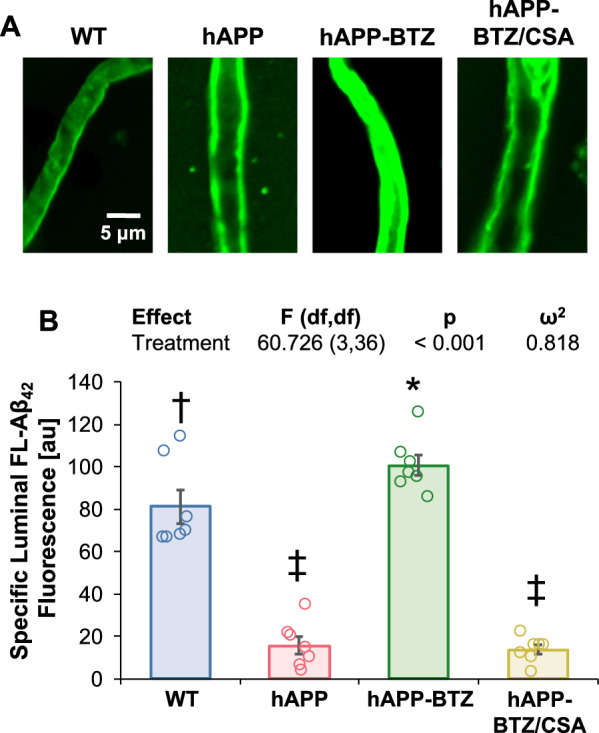
BTZ effect on P-gp-mediated Aβ42 transport in brain capillaries from hAPP mice. **A** Representative confocal microscopy images of brain capillaries isolated from WT mice, vehicle-treated hAPP mice, BTZ-treated hAPP mice, and BTZ/CSA-treated hAPP mice showing Fluorescein-β-amyloid1-42 (FL-hAβ42) accumulation in capillary lumens after a 1-h incubation (steady state; 5 μM FL-hAβ42). **B** Data after digital image analysis using ImageJ. Specific FL-hAβ42 fluorescence is the difference between total luminal FL-hAβ42 and FL-hAβ42 fluorescence with the P-gp-specific inhibitor PSC833 (5 μM). Columns represent mean ± SEM for 7 capillaries from one preparation (pooled tissue: WT: n = 20 mice; hAPP: n = 18 mice; hAPP-BTZ: n = 20 mice, hAPP-BTZ/CSA: n = 19 mice). Symbols indicate statistical groupings of pairwise comparisons adjusted for FDR. Columns that share a symbol do not significantly differ from each other

To assess P-gp-mediated hAβ transport at the blood–brain barrier, we incubated freshly isolated brain capillaries for 1 h with 5 μM FL-hAβ42 to steady-state, imaged capillaries with confocal microscopy and measured luminal FL-hAβ42 fluorescence with digital image analysis (Fig. 3A**)**. Image analysis shows reduced luminal FL-hAβ42 fluorescence in capillaries from hAPP mice (81% signal reduction; Hedges’ *g* = − 3.752) compared to capillaries isolated from WT mice (Fig. 3B). Treating hAPP mice with BTZ brought FL-hAβ42 fluorescence to levels *above* those seen in brain capillaries from WT mice (24% signal *enhancement* vs. WT; Hedges’ *g* = 1.066). We acknowledge a potential ceiling effect for signal detection in the hAPP-BTZ group. Our data suggest that treating hAPP mice with BTZ restored P-gp-mediated hAβ transport in brain capillaries.

### Bortezomib effect in hAPP mice in vivo

**Fig. 4:**
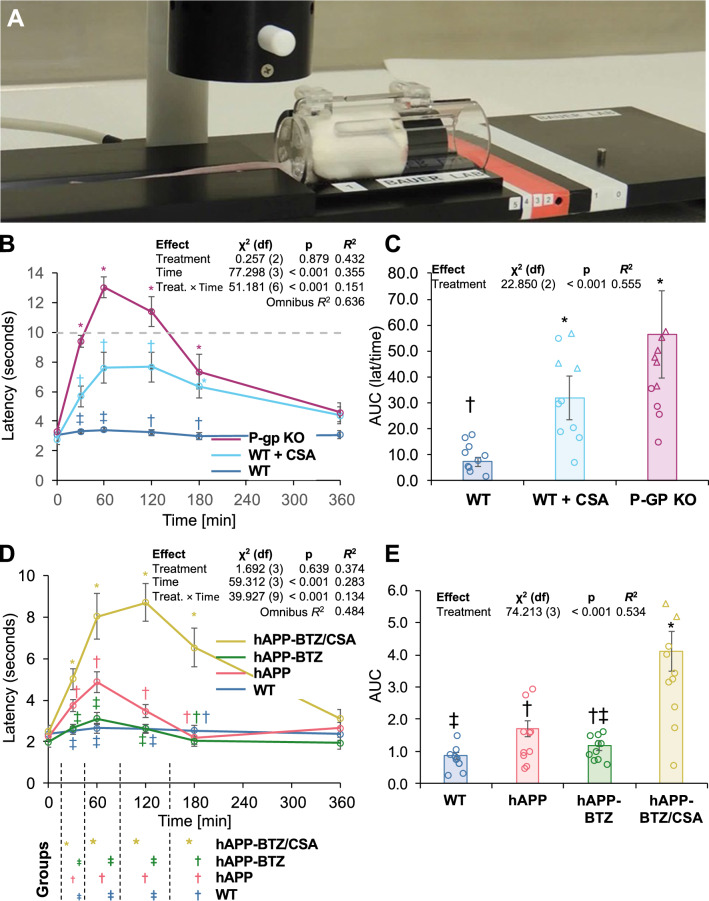
BTZ effect on P-gp transport-dependent nociception in hAPP mice in vivo. **A** Picture of a mouse placed on the LE 7106 Light Beam analgesia meter (Harvard Apparatus, Holliston, MA, USA). **B** Tail flick latency times at 30, 60, 120, 180, and 360 min after loperamide dosing (5 mg/kg; s.c.); maximum possible effect (MPE%) plotted against time. WT mice (n = 10) flicked their tails shortly after the radiant stimulus was applied, indicating that loperamide has no central antinociceptive effect. P-gp KO mice (n = 9) and WT that received the P-gp inhibitor CSA (50 mg/kg; i.p.; n = 11) displayed high MPE% values indicating loperamide had a central antinociceptive effect in these animals. Symbols indicate statistical groupings of pairwise comparisons adjusted for FDR (false discovery rate) at each time point. No symbols indicate no significant differences among any treatments. Points that share a symbol do not significantly differ from each other. **C** The area under the curve (AUC) for the time course was fourfold higher in CSA-treated WT mice and eightfold higher in P-gp KO mice compared to WT mice. The difference in AUCs for P-gp KO mice and CSA-treated WT mice was not statistically significant, although both significantly differed from WT mice. Symbols indicate statistical groupings of pairwise comparisons adjusted for FDR. Columns that share a symbol do not significantly differ from each other. **D** The MPE% in WT mice and BTZ-treated hAPP mice was comparable, whereas hAPP mice and hAPP mice treated with BTZ-CSA had significantly higher MPE% values. Symbols indicate pairwise comparisons adjusted for FDR at each time point (30, 60, 120, and 180 min). Points that share a symbol do not significantly differ from each other. **E** The AUC between WT and BTZ-treated hAPP mice was comparable. AUC values for hAPP mice were significantly higher than those of WT mice. The highest AUC value was observed for hAPP mice treated with BTZ/CSA. Symbols indicate statistical groupings of pairwise comparisons adjusted for FDR. Columns that share a symbol do not significantly differ from each other. Data per group are shown as mean ± S.E.M for each animal: WT: n = 10, hAPP: n = 10, hAPP-BTZ: n = 10, hAPP-BTZ + CSA: n = 10

The tail flick assay is an in vivo test to assess nociception in mice. In this assay, the animal is restrained, and a high-intensity light beam is aimed at its tail, which responds to the heat stimulus with a flick of its tail (Fig. 4A). The meter automatically records the time from the onset of the heat exposure until the mouse flicks its tail. In the present study, we used this test in combination with the P-gp substrate loperamide to indirectly measure P-gp transport activity at the blood–brain barrier in vivo [41]. Loperamide is an over-the-counter antidiarrheal opioid that reduces gut motility by binding to intestinal μ-opioid receptors [34, 42]. Even though loperamide is an opioid, it has no central analgesic effect because it is a P-gp substrate and does not cross the blood–brain barrier [41, 43, 44]. However, when blood–brain barrier P-gp is impaired, loperamide can enter the brain and elicit central antinociception, which can be measured with the tail flick assay. Thus, we used this assay to indirectly measure P-gp transport activity at the blood–brain barrier in vivo.

In a first set of experiments (Fig. 4B, C), we compared tail flick latency times from WT mice (n = 10), WT mice dosed with the P-gp inhibitor CSA (50 mg/kg; i.p.; n = 11), and P-gp KO mice (n = 9); mice from all three groups received 5 mg/kg loperamide (s.c.). Time course analysis revealed significant pairwise differences among treatments at specific time points (Fig. 4B). Between 30 and 180 min (inclusive), the latency for both P-gp KO mice was significantly higher (p ≤ 0.05) than the latency recorded for WT + CSA, which was significantly higher than WT mice, indicating an increased central antinociceptive effect of loperamide effect in animals where P-gp is inhibited (CSA treatment) or lacking (P-gp KO). We also analyzed the area under the curve (AUC) and found that CSA-treated WT mice had a ~ 4.4-fold higher AUC compared to WT mice (Fig. 4C; Table 2). The AUC for P-gp KO mice was 7.8-fold higher compared to that for WT mice. The AUC for each treatment differed significantly (p ≤ 0.05) from each other. These data show that P-gp inhibition or lack of P-gp at the blood–brain barrier results in a central antinociceptive loperamide effect compared to WT controls. This finding set the stage for BTZ treatment studies of hAPP mice.

**Table 2:** AUC values for tail flick assay shown in Fig. 4B

Group	AUC	Fold^a^	*g*^b^
WT	0.110 ± 0.020	1.00	0.00
WT + CSA	0.379 ± 0.062	3.43	1.76
P-gp KO	0.524 ± 0.065	4.75	2.61

^a^AUC ÷ AUC_WT_

^b^Hedges’ *g* vs WT

To assess the BTZ effect on P-gp transport activity in vivo in hAPP mice, we conducted tail flick assays after the two-week dosing regimen with BTZ. Prior to the tail flick assay, mice were injected with the P-gp substrate loperamide (5 mg/kg; s.c.). Figure 4D shows the time course of the loperamide-induced central antinociceptive effect. Note that interaction of treatment and time was significant (*p* < 0.001) overall. When we considered each time point in succession, we found that WT and BTZ-treated hAPP mice did not significantly differ from each other at any time point, suggesting that P-gp at the blood–brain barrier limited loperamide brain uptake, and thus, central antinociception in these two groups. In contrast, at 30–120 min, hAPP mice showed a higher antinociceptive effect than both WT and BTZ-treated hAPP mice. Treating hAPP mice with BTZ/CSA resulted in high antinociception, significantly differing from all other treatments at all time points from 30 to 180 min. Thus, inhibiting P-gp with CSA increased the antinociceptive loperamide effect in the brain. Analysis of AUCs revealed that the AUC for WT mice was lower than that for both hAPP and BTZ/CSA-treated hAPP mice (Fig. 4E). AUC for BTZ-treated hAPP mice was intermediate between WT and hAPP but did not significantly differ from each other. Interpretation of these results is not trivial. Treatment of hAPP mice with BTZ did not reduce AUC to significantly less than for untreated hAPP mice. However, this does not mean that BTZ treatment was without efficacy. Untreated hAPP mice had a significantly higher AUC compared to WT mice, indicating that the hAPP condition elevated nociception. Treatment with BTZ reduced AUC to be statistically indistinguishable (*p* > 0.05) from WT, thus indicating that hAPP + BTZ produced outcomes could not be excluded from being within the limits of WT, even if the effect did not indicate that it differed from untreated hAPP. Treatment with BTZ/CSA resulted in high AUC due to P-gp inhibition.

Together, these data indicate that BTZ protected P-gp transport activity in hAPP mice in vivo*,* resulting in a central antinociceptive effect comparable to that measured in WT mice. This finding suggests that P-gp is functional and restricts loperamide entry into the brain.

### Bortezomib effect on proteasome activity

**Fig. 5:**
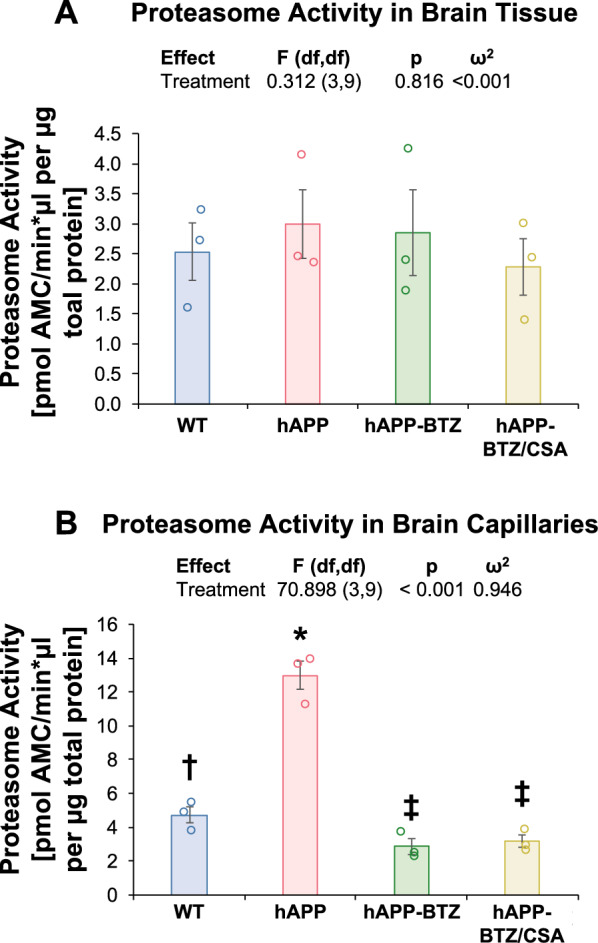
BTZ effect on 20S proteasome activity levels in brain tissue and isolated capillaries. **A** Proteasome activity levels in brain tissue samples from WT, hAPP, hAPP-BTZ, and hAPP-BTZ/CSA mice. Brains were processed and assayed as described in the main text. No significant differences were found among the groups. **B** Increased proteasome activity levels in capillaries isolated from hAPP mice compared to capillaries from WT, hAPP-BTZ, and hAPP-BTZ/CSA mice. Samples were prepared and assayed as described in the text. For all assays, n = 3 per sample

We also determined the BTZ effect on proteasomal activity in brain tissue and isolated brain capillaries. When we measured proteasome activity in brain samples collected from each group (WT, hAPP, hAPP-BTZ, hAPP-BTZ/CSA), we found no difference among the four groups (Fig. 5A). In contrast, measuring proteasome activity in brain capillaries isolated from each group revealed a significant adjusted pairwise difference (*p* < 0.001, + 373%, Hedges’ *g* =  + 5.977) between capillaries from hAPP mice compared to BTZ-treated hAPP mice (Fig. 5B). No difference existed between the proteasome activity in capillaries isolated from WT, BTZ-treated hAPP, or BTZ/CSA-treated hAPP mice. Thus, proteasome activity was elevated in capillaries from hAPP mice, and BTZ reversed this effect.

### Bortezomib effect on Aβ levels in hAPP mice

**Fig. 6:**
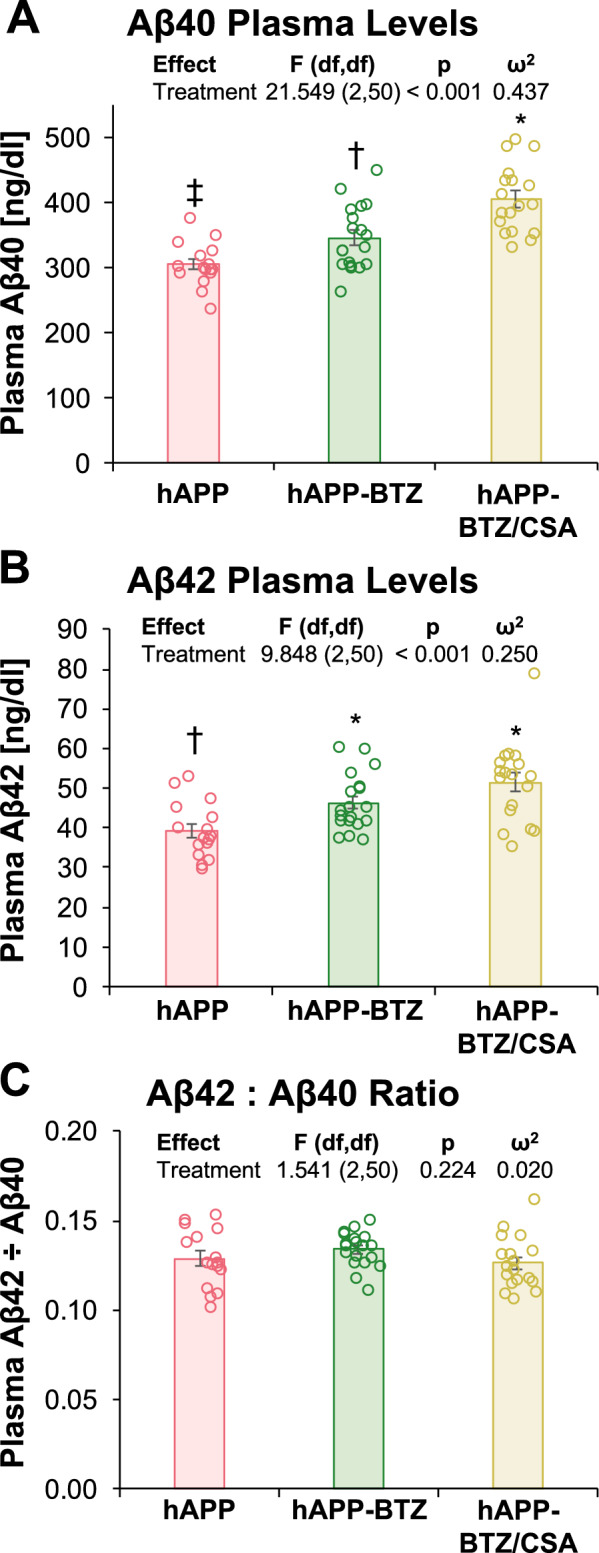
BTZ effect on Aβ40 and Aβ42 plasma levels in hAPP mice. **A** Aβ40 (pg/ml) and **B** Aβ42 plasma levels (ng/dl) in samples from hAPP, hAPP-BTZ, and hAPP-BTZ/CSA mice. The overall tendency was for hAPP mice to have the lowest Aβ40 and Aβ42 plasma levels, which was significant for both Aβ forms. The difference between BTZ and BTZ/CSA was only significant for Aβ40. **C** Aβ42/Aβ40 ratios. No significant differences appeared by treatment. Symbols indicate statistical groupings of pairwise comparisons adjusted for FDR. Columns that share a symbol do not significantly differ from each other. hAPP: n = 16, hAPP-BTZ: n = 19, hAPP-BTZ + CSA: n = 18

Next, we determined the BTZ effect on plasma Aβ40 or Aβ42 levels in hAPP mice. WT mice do not express human Aβ and therefore were not tested. To assess Aβ40 and Aβ42 levels, we performed Aβ40- and Aβ42-specific ELISA using plasma collected from hAPP mice. Mean Aβ40 levels were 305 ± 8 ng/dl for hAPP mice, 346 ± 11 ng/dl for hAPP-BTZ mice, and 406 ± 12 ng/dl for hAPP-BTZ/CSA mice (Fig. 6A). Pairwise testing revealed that Aβ40 in each treatment significantly differed, indicating that BTZ and BTZ/CSA treatment increased Aβ40 plasma levels. Mean Aβ42 levels were 39.4 ± 1.7 ng/dl for hAPP mice, 46.4 ± 1.6 ng/dl for hAPP-BTZ mice, and 51/6 ± 2.4 ng/dl for hAPP-BTZ/CSA mice (Fig. 6B). Aβ42 plasma levels were significantly higher in BTZ and BTZ/CSA (grouped) vs. vehicle-treated hAPP mice. Aβ42/Aβ40 ratios were 0.129 ± 0.004, 0.134 ± 0.002, and 0.126 ± 0.003, respectively; thus, BTZ had no overall significant effect on Aβ42/Aβ40 ratios (Fig. 6C).

**Fig. 7:**
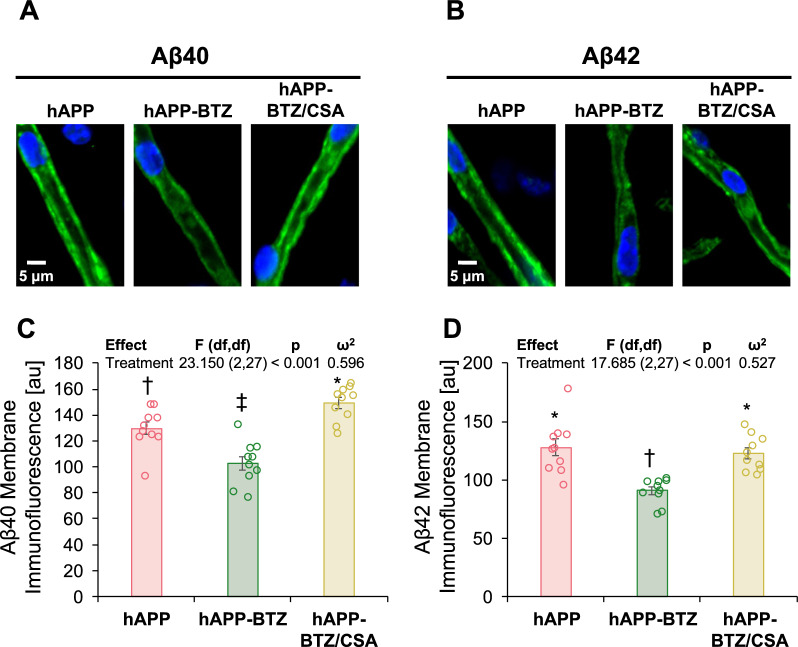
BTZ effect on capillary Aβ levels. Representative confocal microscopy images of isolated brain capillaries immunostained for **A** Aβ40 (green: Aβ40; blue: nuclei counterstained with DAPI) and **B** Aβ42 (green: Aβ40; blue: nuclei counterstained with DAPI). Data after digital image analysis of membrane-associated **C** Aβ40- and **D** Aβ42-immunofluorescence using ImageJ. Statistics: Data per group are given as mean ± SEM for 10 capillaries from one preparation (pooled tissue: WT: n = 10 mice, hAPP: n = 15 mice, hAPP-BTZ: n = 15 mice, hAPP-BTZ + CSA: n = 15 mice). Shown are arbitrary fluorescence units (scale 0–255). Symbols indicate statistical groupings of pairwise comparisons adjusted for FDR. Columns that share a symbol do not significantly differ from each other

To determine capillary-associated Aβ deposition, we isolated brain capillaries from hAPP mice and immunostained them for Aβ40 and Aβ42. Capillaries were imaged with a confocal microscope, and Aβ40 and Aβ42 immunofluorescence was digitally analyzed with ImageJ. Brain capillaries from hAPP mice of all groups stained positive for Aβ40 or Aβ42 (Fig. 7A-B). Negative controls for each treatment were processed without primary antibody and showed negligible background fluorescence (data not shown). We found that capillary-associated Aβ40 levels were 21% lower, and Aβ42 levels were 29% lower in BTZ-treated hAPP mice compared to untreated hAPP mice (Figs. 6C and 6D). Pairwise comparisons indicated that Aβ40 levels from BTZ-treated hAPP mice were significantly (*p* ≤ 0.05) lower than those for vehicle-treated hAPP mice and lower than that from hAPP-BTZ/CSA-treated mice. For Aβ40, levels from BTZ-treated hAPP mice were significantly (*p* ≤ 0.05) lower than those for vehicle-treated hAPP mice. Thus, BTZ treatment reduced capillary-associated Aβ deposition in hAPP mice.

**Fig. 8:**
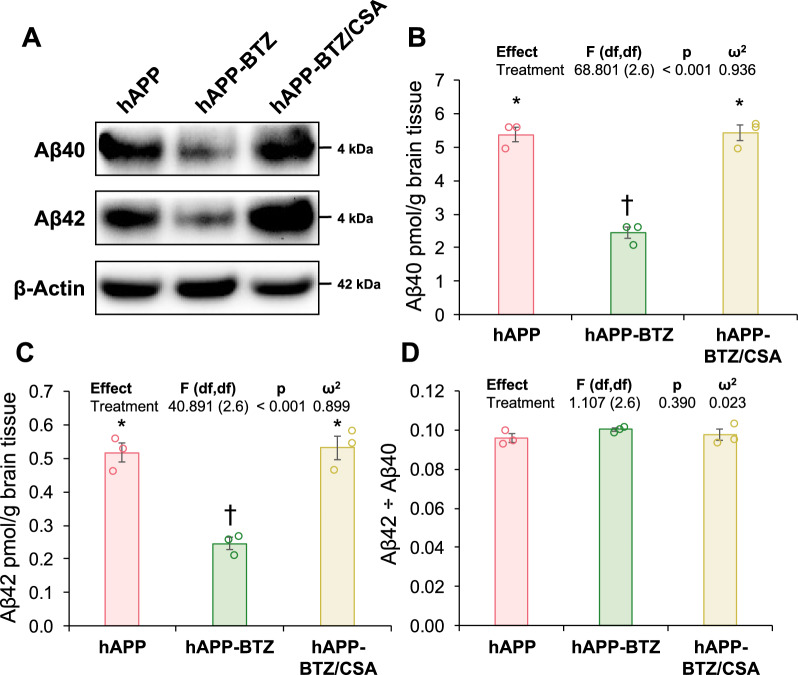
BTZ effect on Aβ brain levels. **A** Western blot showing Aβ40 and Aβ42 protein expression levels in brain tissue samples from vehicle-treated hAPP mice, hAPP mice dosed with 0.25 mg/kg BTZ, and hAPP mice dosed with a combination of BTZ and CSA (0.25/25 mg/kg). β-Actin was used as protein loading control. **B** Aβ40 and **C** Aβ42 levels in pg/g brain tissue in brain tissue samples from hAPP, hAPP-BTZ, and hAPP-BTZ/CSA mice determined by ELISA. **D** Aβ42/Aβ40 ratio. Symbols indicate statistical groupings of pairwise comparisons adjusted for FDR. Columns that share a symbol do not significantly differ from each other

We also analyzed Aβ40 and Aβ42 in brains by Western blotting and ELISA. Western blotting data showed decreased Aβ40 and Aβ42 protein levels in brain tissue samples from BTZ-treated hAPP mice compared to untreated and BTZ/CSA-treated hAPP mice (Fig. 8A). To quantify Aβ brain levels, we used ELISA and found that vehicle-treated hAPP mice had 5.385 ± 0.215 pmol Aβ40/g brain tissue, BTZ-treated hAPP mice 2.447 ± 0.180 pmol/g, and BTZ/CSA-treated hAPP mice 5.436 ± 0.230 pmol/g (Fig. 8B). Aβ42 brain levels were 0.518 ± 0.029, 0.246 ± 0.018, and 0.532 ± 0.034 pmol/g brain tissue, respectively (Fig. 8C). Ratios were similar at 0.096 ± 0.002 for hAPP, 0.101 ± 0.0001 for hAPP-BTZ, and 0.098 ± 0.005 for hAPP-BTZ/CSA hAPP mice (Fig. 8D**; **Table 3). When analyzed for pairwise differences, we found that hAPP mice treated with BTZ had significantly (*p* ≤ 0.05) lower Aβ40 and Aβ42 brain levels than vehicle-treated hAPP mice and BTZ/CSA-treated hAPP mice. Thus, BTZ treatment significantly reduced Aβ brain levels compared to untreated hAPP mice. In contrast, Aβ brain levels remained unchanged in BTZ-treated hAPP mice that also received the P-gp inhibitor CSA.

**Table 3:** Effect sizes of hAβ42/hAβ40 ratio tests

Comparison	Serum ELISA		Capillaries		Brain ELISA	
	%	*g*	%	*g*	%	*g*
hAPP vs hAPP-BTZ	4	0.367	11	0.491	5	0.980
hAPP vs hAPP-BTZ/CSA	2	0.193	22	0.854	2	0.368
hAPP-BTZ vs hAPP-BTZ/CSA	6	0.559	9	0.363	3	0.612

Together, these data suggest that treating hAPP mice with BTZ protects P-gp from proteasomal degradation, which reduces Aβ levels in capillaries and brain tissue.

## Discussion

We previously demonstrated that human Aβ40 triggers P-gp ubiquitination, internalization, and proteasomal degradation resulting in reduced blood–brain barrier P-gp protein expression and transport activity in isolated brain capillaries [25]. We also showed that treating hAPP mice with the ubiquitination inhibitor PYR41 prevents P-gp ubiquitination and degradation in vivo [26]. In a follow-up study, blocking P-gp internalization with the microtubule inhibitor nocodazole (NCZ) maintained P-gp protein expression and transport activity levels in isolated capillaries from NCZ-treated hAPP mice [24]. In both in vivo studies, preventing loss of P-gp by targeting the ubiquitin–proteasome pathway protected blood–brain barrier P-gp, which helped to reduce Aβ brain levels in hAPP mice. The present study extends these findings by inhibiting the 20S proteasome with the FDA-approved drug bortezomib (BTZ) to protect P-gp from proteasomal degradation in hAPP mice in vivo.

**Fig. 9:**
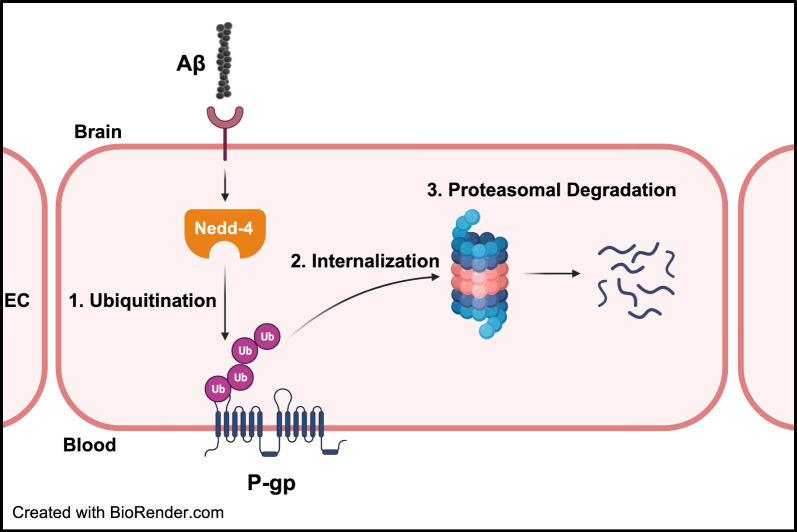
Proposed mechanism. Our current findings and our previously published data [24–26, 71] suggest that Aβ (1) activates the ubiquitin ligase NEDD4 [71], which (2) tags the transporter with ubiquitin [25, 26], a signal that leads to (3) internalization of P-gp followed [24] by (4) proteasomal degradation (present study). In this series of three independent in vivo studies, we showed that when we pharmacologically intervene by blocking (1) ubiquitination, (2) internalization, and (3) proteasomal degradation of P-gp we prevent P-gp loss at the blood–brain barrier, which in turn lowers Aβ brain levels in vivo [24, 26], as we show herein. Created with BioRender.com

We treated 10–11-week-old hAPP mice with BTZ for two weeks (Fig. 1**; **Table 1). At this age, hAPP-transgenic mice exhibit Aβ brain accumulation but do not show cognitive impairment yet, thus representing a model for early-stage AD pathogenesis that includes blood–brain barrier dysfunction [9, 24, 35, 45–47]. We found that P-gp protein and transport activity levels in isolated brain capillaries from BTZ-treated hAPP mice were comparable to levels detected in control WT mice (Fig. 2). In addition, we observed a similar BTZ effect on P-gp-mediated Aβ transport that was restored to control levels in capillaries from BTZ-treated hAPP mice (Fig. 2). Our data confirmed that capillaries from hAPP mice have elevated proteasome activity and that BTZ treatment reduced these to WT levels (Fig. 4). Using the tail flick assay, we found that hAPP mice had a significantly higher loperamide-induced central antinociceptive response compared to untreated WT mice (Fig. 5). In contrast, BTZ treatment reversed this effect, indicating that BTZ protected P-gp at the blood–brain barrier in hAPP mice in vivo and prevented loperamide from getting into the brain, inducing central antinociception. In contrast to our previous studies, we found elevated Aβ40 and Aβ42 plasma levels in response to BTZ treatment (Fig. 6). CSA co-treatment increased this effect. CSA is not a specific P-gp inhibitor, therefore treatment with CSA could also lead to the inhibition of Oatp1a4 or other ABC transporters implicated in Aβ clearance [62–64]. Future studies are needed to determine the effect of BTZ on Oatp1a4 and other ABC transporters. In contrast to Aβ plasma levels, proteasome inhibition significantly lowered Aβ40 and Aβ42 levels in capillary membranes and brain tissue from BTZ-treated hAPP mice (Fig. 7–8). No significant changes were found in the Aβ42/Aβ40 ratio. We acknowledge that no brain specific Aβ measurements were taken. Based on these data we conclude that the FDA-approved inhibitor BTZ protects P-gp from proteasomal degradation resulting in reduced Aβ capillary and brain levels in hAPP mice (Fig. 9). In the following sections, we highlight limitations of our study and discuss our findings in the context of the existing literature.

### Limitations of the study

The present study belongs to a series of three independent in vivo studies showing that when we pharmacologically intervene by blocking (1) ubiquitination [26], (2) internalization [24], and (3) proteasomal degradation of P-gp (present study), we prevent P-gp loss at the blood–brain barrier, which in turn lowers Aβ brain levels in vivo*.* For all three studies we used young mice to establish the proof-of-concept that P-gp can be protected by blocking the ubiquitin–proteasome pathway. Since our three initial studies were successful, we have critical information needed to plan an informative long-term study to test the effect of BTZ on slowing cognitive decline. Since cognitive dysfunction is the fundamental clinical trait of AD patients, future investigation using aged mice is needed. Further, for these proof-of-concept studies we chose male mice to avoid any hormone-dependent effects that could interfere with our study. Since sex differences exist in patients, future studies focusing on cognitive decline should include both sexes.

### Potential benefit of BTZ for AD therapy

The FDA-approved proteasome inhibitor BTZ is widely used to treat multiple myeloma mantle cell lymphoma. BTZ is a boronic acid dipeptide derivative with C_max_ of 106.2 ± 46.7 ngh/ml and a half-life (t_½_) of 78.9 ± 50.9 h [65]. Treatment is by intravenous injection, with recommendation to not use any other route. Potential unwanted effects include peripheral neuropathy (up to Grade 4), hypotension, posterior reversible encephalopathy syndrome, thrombocytopenia/neutropenia, and multiple toxicities (cardiac, pulmonary, gastrointestinal, and hepatic), among several other adverse reactions. Eleven percent of patients have been reported to experience severe toxicity (greater than Grade 4) [66]. BTZ is an anticancer drug, where the need for life-saving measures can override patient discomfort in the short term. Given its severe side effects, it may not be appropriate to use specifically as an AD treatment, particularly not if chronically administered. However, subcutaneous administration of BTZ may reduce neuropathy [67], and related class drugs, including Ixazomib and Marizomib, produce a lower incidence and severity of side effects [67–70]. Thus, although our use of BTZ illustrates an important pathway that can be targeted to reduce Aβ brain load, therapeutic application of the current class and generations of proteasome inhibitors may not be appropriate for AD, considering quality-of-life and the current consensus that effective treatment of AD would need to be given in the early or even prodromal stages, where symptoms have not yet become severe.

### Loss of P-gp at the blood–brain barrier in AD

The loss of blood–brain barrier P-gp in AD is well-documented: data from several studies show that P-gp protein expression levels are significantly reduced at the blood–brain barrier in AD patients [14, 15, 17, 18, 22]. For example, using automated image analysis of brain sections, investigators at the NIH and the Karolinska Institute reported a 53% reduction (*p* < 0.01) in P-gp protein levels in capillaries from AD patient brain samples compared to samples from control individuals [48]. Consistent with reduced P-gp expression levels, results of PET studies indicated compromised P-gp activity in AD patients [16, 21]). Overall, the existing studies support the conclusion that blood–brain barrier P-gp is significantly reduced in AD.

Here we show for the first time that P-g loss has a consequence for brain uptake of P-gp substrates. Using the tail flick assay, we demonstrate in vivo that the P-gp substrate loperamide has an antinociceptive effect in hAPP mice, suggesting that P-gp loss enabled loperamide to get into the brain and block opioid receptors. These data indirectly show that P-gp transport activity is severely impaired at the blood–brain barrier in vivo in hAPP mice leading to increased brain uptake and pharmacological effect in the brain of P-gp substrates. These results are consistent with our previous findings, where P-gp transport activity was impaired ex vivo in isolated mouse brain capillaries from 12-week-old hAPP transgenic mice [9, 25, 26]. Further, we demonstrate that BTZ-treated hAPP mice did not show an antinociceptive effect after loperamide dosing, suggesting that BTZ protected hAPP mice from loss of P-gp transport function at the blood–brain barrier. Additional dosing with the P-gp inhibitor CSA reversed the BTZ effect, leading to significant antinociception in hAPP mice. This observation is consistent with CSA treatment inhibiting P-gp, which enabled loperamide to cross the blood–brain barrier, further validating our in vivo assay. In summary, by using the tail flick assay, we demonstrate for the first time that P-gp transport function is impaired in vivo in hAPP transgenic mice long before cognitive decline is apparent.

### P-gp protein expression and activity are regulated through the ubiquitin–proteasome pathway

In 1998, Labroille and coworkers [49] were the first to propose P-gp trafficking between a cytoplasmic P-gp pool and the cell membrane is important to maintain a steady-state level of surface P-gp in cells. Loo and Clarke [50] reported that P-gp trafficking to the cell surface depends on transmembrane domains TMD1 and TMD2. In addition, they found that the proteasome is involved in the degradation of misprocessed mutants of P-gp [51]. In this regard, Gibrar et al. [52] found that the proteasome degrades improperly folded mutant P-gp. Earlier work by Egner and Kuchler [53] showed that the ABC transporter Pdr5, the counterpart of mammalian P-gp in yeast, is ubiquitinated, and ubiquitin modification of the transporter is a signal for degradation by the 26S proteasome. At the time, the authors speculated that ubiquitination of yeast ABC transporters could signal trafficking that would lead to internalization of the transporter, proteasomal degradation, and ultimately turnover of P-gp proteins at the cell surface. Regarding P-gp degradation, several studies have shown that cell surface P-gp in cancer cells is regulated by the 26S proteasome, a complex consisting of a 20S core and a 19S regulatory subunit that degrades ubiquitinated proteins [54–56]. Together, these findings in cancer cell lines and yeast indicate that the ubiquitin–proteasome system regulates P-gp levels and built the scientific foundation for our hypothesis that proteasome inhibition could be one way to protect P-gp from proteasomal degradation. Importantly, our study shows for the first time that proteasome activity is elevated in brain capillaries from hAPP mice compared to WT mice. These elevated activity levels likely contribute to P-gp loss at the blood–brain barrier in AD. In contrast, proteasome activity in brain tissue samples was similar across all groups suggesting that accelerated proteasomal degradation of P-gp is confined to the capillary endothelium, indicating a brain endothelial-specific process. Overall, dysfunction of the ubiquitin–proteasome system is known to play a role in AD and contribute to pathogenesis [57]. Specifically, studies found decreased proteasome activity in the hippocampus, a brain region susceptible to AD pathology. Other brain regions, such as the cerebellum, showed no changes in proteasome activity in samples from AD patients compared to controls [58, 59]. A 50% decrease in proteasome activity was detected in primary neurons isolated from hAPP mice (Tg2576) compared to neurons isolated from wild-type mice [60]. Together these data show that proteasome activity in AD is decreased in some brain regions, whereas activity levels remain unchanged in other areas. Our work suggests that proteasome activity is upregulated at the blood–brain barrier before cognition deficits become apparent. More work is needed to determine activity levels in different brain regions and at the blood–brain barrier throughout the disease in patients.

## Conclusion

Current and previous findings support the hypothesis that P-gp is one critical factor in defective Aβ brain clearance [6, 7, 9, 25, 26, 39]. Thus, P-gp could serve as a potential target to lower Aβ brain levels and, therefore, to slow progression of the disease or even prevent its onset. Our combined data from three independent in vivo studies indicate that the ubiquitin–proteasome system could be a potential therapeutic target to prevent P-gp loss in AD [24–26]. Specifically, we found that the ubiquitination inhibitor PYR-41, the microtubule inhibitor nocodazole, and the proteasome inhibitor BTZ all protected P-gp and lowered hAβ brain levels in transgenic mice with equal efficacy. BTZ (marketed as Velcade^®^) is the most promising compound of these for clinical application since it is already an FDA-approved drug (treatment of refractory multiple myeloma and mantle cell lymphoma) and, thus, could potentially be repurposed for the treatment of AD [61]. However, further investigation is needed to demonstrate that BTZ treatment slows progression of cognitive decline in hAPP mice before clinical trials with BTZ in AD patients should be considered.

## Data Availability

The datasets used and/or analyzed in the current study are available from the corresponding author upon reasonable request.

## Abbreviations

CNS: Central nervous system
PBS: Phosphate buffered saline
TBS: Tris-buffered saline
hAβ: Human amyloid beta
P-gp: P-glycoprotein
